# CircFunBase: a database for functional circular RNAs

**DOI:** 10.1093/database/baz003

**Published:** 2019-02-04

**Authors:** Xianwen Meng, Dahui Hu, Peijing Zhang, Qi Chen, Ming Chen

**Affiliations:** 1 Department of Bioinformatics, the State Key Laboratory of Plant Physiology and Biochemistry, Institute of Plant Science, College of Life Sciences, Zhejiang University, Hangzhou, China; 2 The State Key Laboratory of Crop Biology, Shandong Key Laboratory of Crop Biology, College of Agronomy, Shandong Agricultural University, Tai'an, China

## Abstract

Increasing evidence reveals that circular RNAs (circRNAs) are widespread in eukaryotes and play important roles in diverse biological processes. However, a comprehensive functionally annotated circRNA database is still lacking. CircFunBase is a web-accessible database that aims to provide a high-quality functional circRNA resource including experimentally validated and computationally predicted functions. The current version of CircFunBase documents more than 7000 manually curated functional circRNA entries, mainly including *Homo sapiens*, *Mus musculus* etc. CircFunBase provides visualized circRNA-miRNA interaction networks. In addition, a genome browser is provided to visualize the genome context of circRNAs. As a biological information platform for circRNAs, CircFunBase will contribute for circRNA studies and bridge the gap between circRNAs and their functions.

## Introduction

Circular RNAs are a special group of endogenous RNAs characterized by covalent closed loops (1). CircRNAs are identified to be widespread in both animals and plants through high-throughput sequencing technology coupled with bioinformatics analysis (2–5). Although their biological functions remain largely unknown, increasing evidence suggests that they play important roles in the regulation of multiple biological processes, especially in human diseases (6, 7). One surprising finding is that a novel circular RNA, ciRS-7, could function as a designated miR-7 sponge (8, 9), and, by this way, ciRS-7 might be correlated with human cancers (10–12).

New circRNA detection tools are constantly being developed (13). Together with high-throughput sequencing technologies, thousands of circRNAs in both animals and plants have been reported and integrated into the circRNA databases, such as circBase (14), CIRCpedia (15) and PlantCircNet (16) (Table 1). Although these circRNA databases provide the tissue information, the functions of circRNAs in a specific tissue are not clear. Circ2Traits associates circRNAs with human diseases based on the interactions of circRNAs with disease-associated miRNAs and disease-associated single nucleotide polymorphisms mapped on circRNA loci. However, these potential associations of circRNAs with diseases in human remain to be validated experimentally. Although CircR2Disease is a manually database, it is limited to the circRNAs in the context of diseases.

**Table 1:** Web-based resource for circRNAs

**Name**	**Sample**	**Description**	**Address**	**Reference**
circBase	Animals	A unified circRNA data set from previous publications	http://circbase.org	(14)
CIRCpedia	Animals	CircRNA annotations from over 180 RNA-seq data sets across six different species	http://www.picb.ac.cn/rnomics/circpedia	(15)
CircR2Disease	Animals	Experimentally supported associations between circRNAs and diseases	http://bioinfo.snnu.edu.cn/CircR2Disease	(17)
Circ2Traits	Human	CircRNAs potentially associated with disease and traits	http://gyanxet-beta.com/circdb	(18)
CircInteractome	Human	Interacting miRNAs and RNA-binding proteins of circRNAs	https://circinteractome.nia.nih.gov	(19)
CircNet	Human	CircRNA-miRNA-mRNA interaction networks	http://syslab5.nchu.edu.tw/CircNet	(20)
circRNADb	Human	CircRNAs with protein-coding potential	http://reprod.njmu.edu.cn/circrnadb	(21)
CSCD	Human	Cancer-specific circRNAs	http://gb.whu.edu.cn/CSCD	(22)
TSCD	Human and mouse	Tissue-specific circRNAs	http://gb.whu.edu.cn/TSCD	(23)
PlantcircBase	Plants	Plant circRNAs	http://ibi.zju.edu.cn/plantcircbase	(24)
PlantCircNet	Plants	Providing plant circRNA-miRNA-gene regulatory networks, as well as circRNA information and circRNA expression profiles	http://bis.zju.edu.cn/plantcircnet	(16)

The number of publications about circRNA research keeps increasing rapidly in recent years (25), and the findings from these publications are essential for further studying circRNA functions. To bridge the gap between circRNAs and their functions, we collect current findings about circRNA functions from literature and form a unique functional circRNA resource (CircFunBase, http://bis.zju.edu.cn/CircFunBase). The current version of CircFunBase contains more than 7000 manually curated functional circRNAs, involving 15 organisms (such as *Homo sapiens* and *Mus musculus*). Hence, CircFunBase serves as a more specific functional circRNA resource to efficiently investigate, browse a particular circRNA and provide insights into its function. These functional circRNAs can be easily queried and downloaded through the webpage. In addition, CircFunBase allows researchers to submit novel functional circRNAs.

### Materials and methods

In order to collect all functional circRNAs, we screened all of the literature in the PubMed database with the following keywords: ‘circular RNA’, ‘circRNA’ or ‘RNA circularization’. The relevant hits were downloaded and further inspected manually. We extracted information on functional circRNAs, their related diseases and biological regulations. The GO annotations were downloaded from the Gene Ontology Consortium (26). Besides, the RNA-binding proteins (RBPs) matching to human functional circRNAs were from CircInteractome (19), which provides a comprehensive binding map of RBPs to circRNA using cross-linking immunoprecipitation data. Mature miRNA sequences were acquired from miRBase (27), while animal and plant miRNA-circRNA interactions were predicted by miRanda (28) and TargetFinder (29), respectively.

The CircFunBase database is implemented using HTML and PHP languages with MySQL. The interface component consists of web pages designed and implemented in HTML/CSS. Cytoscape.js (30) was used to visualize the circRNA-associated networks, while Dalliance (31) was used to view the genome. The BLAST module was implemented using SequenceServer (32).

**Figure 1:**
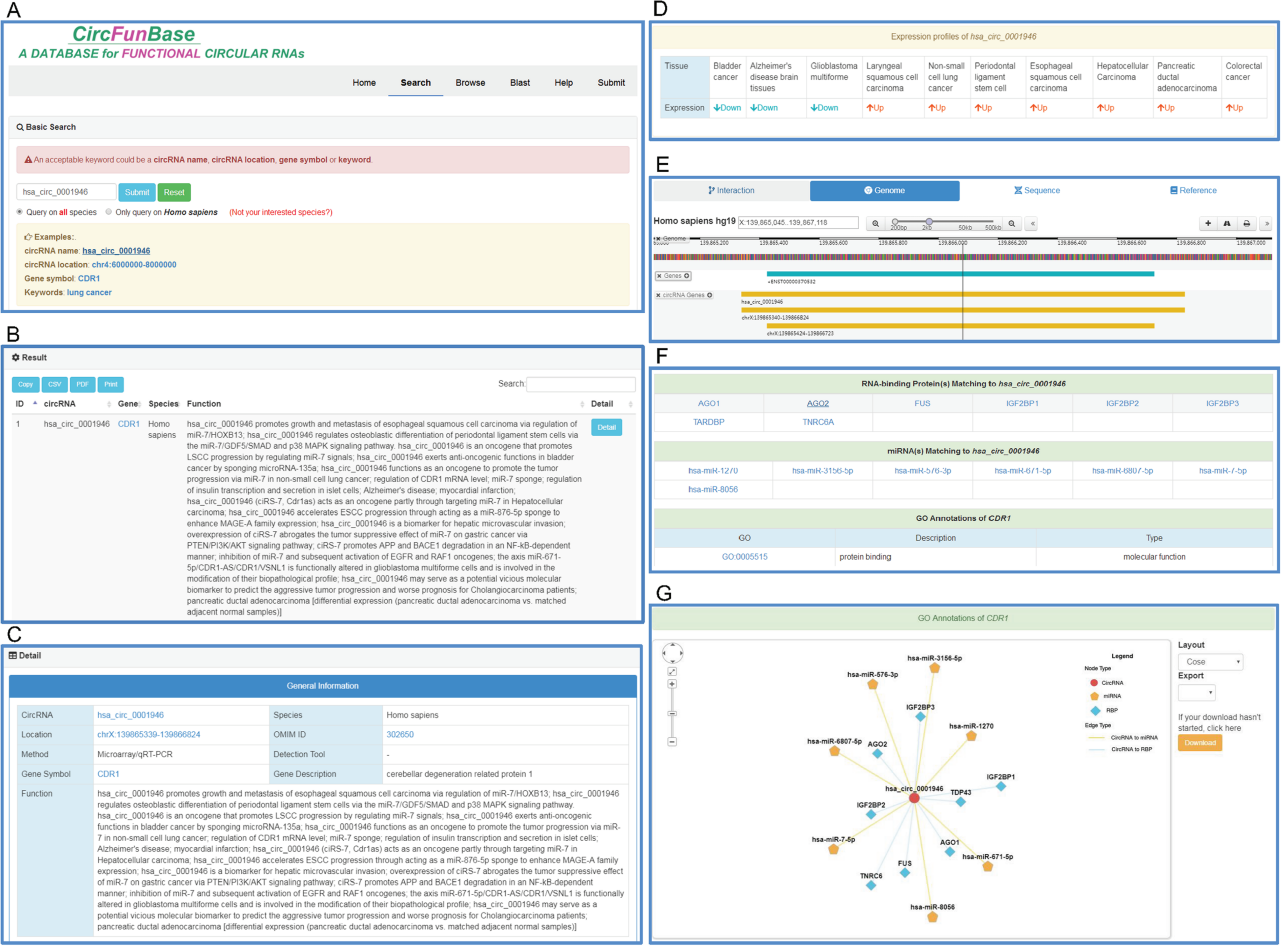
An overview of the user interface of CircFunBase. (A) The search page of CircFunBase. (B) Hsa_circ_0001946 as an example of input and the search result. (C) Detailed information of hsa_circ_0001946. (D) The expression pattern of hsa_circ_0001946. (E) Visualization of the circRNA genome location. (F) CircRNA-associated interactions and GO annotations of parent genes. (G) Visualization of circRNA-associated interactions.

## Results

### Database contents

Functional circRNAs were manually obtained from articles published in the PubMed database before 1 May 2018. In the current version, CircFunBase documents 7059 functional circRNA entries from 15 organisms, including 7 plants and 8 animals. Each entry contains circRNA name, position, tissue, expression pattern, detection tool, function, gene symbol, gene description, PubMed ID, GO annotations and circRNA-associated miRNAs. Particularly, for human, the OMIM ID of parent gene and circRNA-associated RBPs are provided. In addition, a network viewer is used to visualize miRNA-circRNA and RBP-circRNA interactions, and a genome browser is used to view the circRNA genome. Figure 1 illustrates an overview of the user interface of CircFunBase database.

CircFunBase provides the ‘Submit’ page, inviting researchers to upload novel functional circRNAs. In the ‘Help’ page, instructions for using CircFunBase are available and functional circRNA list from each species could be downloaded.

### Data querying, searching and browsing

CircFunBase provides a user-friendly interface for retrieval of functional circRNAs in the ‘Search’ page. Users can retrieve data by circRNA name, circRNA location, gene symbol or keywords (such as ‘lung cancer’ for human). CircFunBase provides brief description of search results in the ‘Search Result’ page. To gain more detail information of a specific circRNA, users can click the ‘Details’ button. Additional information such as PubMed ID, GO annotations and circRNA-associated miRNAs are displayed in the circRNA single-record page. We also provide many useful hyperlinks: (1) human and mouse circRNAs are linked to circBase and could be viewed in UCSC Genome Browser; (2) gene symbol is linked to the NCBI Gene (33) [for *Solanum lycopersicum*, gene symbol is linked to Ensembl (34)]; (3) clicking the GO term links to the GO Consortium website; and (4) miRNA name is linked to mirBase. A BLAST module was provided to query circRNAs in CircFunBase using RNA sequences. In ‘Browse’ page, users can browse all the functional circRNAs in a species by clicking the icon of corresponding species or using the lineage tree. In addition, CircFunBase provides a series of APIs to return detailed information about circRNAs in JSON format, for example, circRNA information or miRNA interaction information.

**Figure 2:**
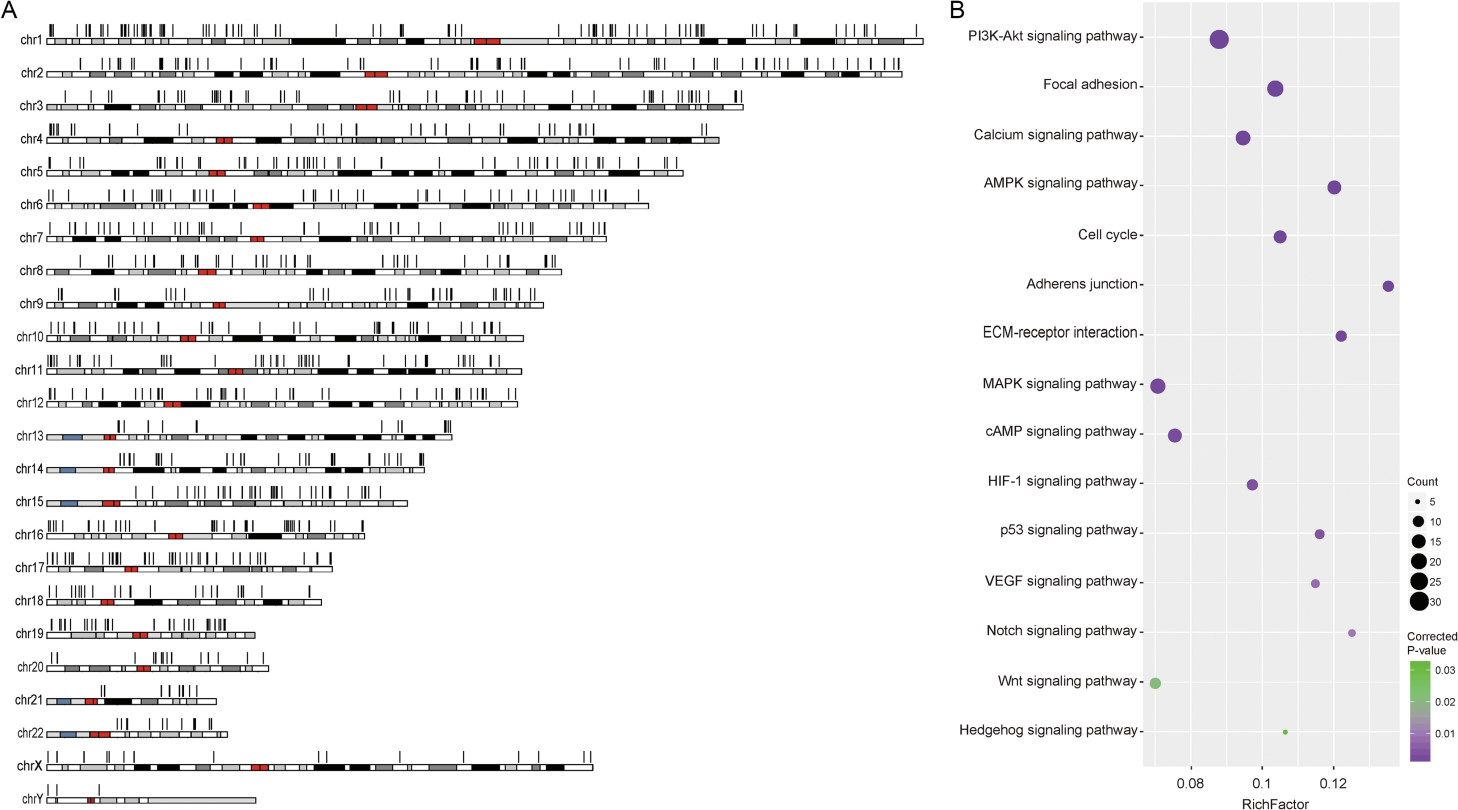
Human cancer circRNAs. (A) Genomic distribution of human cancer circRNAs. (B) Enriched KEGG pathways of parent genes of cancer circRNAs.

### The parent genes of cancer circRNAs are highly correlated with cancer pathways

To highlight the significance of CircFunBase, we presented a biological application based on this data resource. During data collection, we found that a large amount of human circRNAs were correlated to cancers. Previous studies have revealed that most human circRNAs are generated from exon regions of protein-coding genes. Whether the parent genes share similar functions with cancer circRNAs remains to be elucidated. Therefore, we explored this issue using the circRNAs annotated in CircFunBase.

First, we retrieved human cancer circRNAs in CircFunBase using the keyword `cancer’, and we got 1712 cancer circRNAs—of which, 92.87% circRNAs are exonic circRNAs (Figure 2A). Particularly, most cancer circRNAs (96.14%) were identified according to their differential expression in the context of cancer and the functional relationship between circRNAs and their parent genes remains to be investigated. Then, we performed functional enrichment analysis on the parent genes of these cancer circRNAs using KOBAS (35). The results showed that the parent genes of cancer circRNAs were highly correlated with cancer since most of them were significantly enriched in cancer-related pathways (Figure 2B). For example, both circITCH and ITCH were implicated in human cancers by regulating the Wnt signalling pathway (36, 37). Thus, the circRNAs share similar functions with their parent genes in the context of human cancer, and the circRNA functions in cancers can be predicted according to the functions of their parent genes. Actually, circRNA study provides new insights into the mechanisms of human diseases.

## Discussion

As a comprehensive functional circRNA database, CircFunBase is designed to provide a rich data resource for circRNA study. CircFunBase has collected recently discovered functional circRNAs from relevant literature. Currently, more than 7000 functional circRNAs from 15 model species are deposited in CircFunBase. About 75.83% human circRNAs annotated in CircFunBase overlap with the current circRNA reference database, circBase. As circBase does not provide functional information of circRNAs, which is available in CircFunBase, our database will definitely help researchers to better understand circRNA biology. To gain insight into circRNA regulations, CircFunBase provides the circRNA associated RBPs and miRNAs. These interactions will benefit further studies on circRNA functions. Take hsa_circ_0001946 for example, except for experimentally validated miR-7-hsa_circ_0001946 and miR-671-hsa_circ_0001946 interactions, other five miRNAs (miR-576, miR-1270, miR-3156, miR-6807 and miR-8056) targeting hsa_circ_0001946 were also identified in CircFunBase, the biological functions of these novel interactions in specific contexts remain to be explored in the future. Another biological application based on the resource provided by CircFunbase revealed that the parent genes of cancer circRNAs are highly correlated with cancer pathways. The functional consistency between parent genes and circRNAs in human cancers is of great significance for exploring the mechanisms of cancers.

To our knowledge, CircFunBase is the first database focusing on circRNA functions in diverse species. It will bridge the gap in circRNAs and functional research and further facilitate biologists in unveiling the roles of circRNAs in diverse biological processes. We will update CircFunBase regularly with newly published data. In addition, direct data submission by the researchers is supported. With the improvement of CircFunBase, it is expected to become a valuable data resource and serve as a foundation for future circRNA study.

## Notes

**Database URL**: http://bis.zju.edu.cn/CircFunBase
